# Electrochemical performance and interfacial properties of Li-metal in lithium bis(fluorosulfonyl)imide based electrolytes

**DOI:** 10.1038/s41598-017-16268-7

**Published:** 2017-11-21

**Authors:** Reza Younesi, Fanny Bardé

**Affiliations:** 1 Department of Chemistry-Ångström Laboratory, Uppsala University, SE-75121, Uppsala, Sweden; 2 Toyota Motor Europe, Research & Development 3, Advanced Technology 1, Hoge Wei 33B, B-1930 Zaventem, Belgium

## Abstract

Successful usage of lithium metal as the negative electrode or anode in rechargeable batteries can be an important step to increase the energy density of lithium batteries. Performance of lithium metal in a relatively promising electrolyte solution composed of lithium bis(fluorosulfonyl)imide (LiN(SO_2_F)_2_; LiFSI) salt dissolved in 1,2-dimethoxyethane (DME) is here studied. The influence of the concentration of the electrolyte salt −1 M or 4 M LiFSI- is investigated by varying important electrochemical parameters such as applied current density and plating capacity. X-ray photoelectron spectroscopy analysis as a surface sensitive technique is here used to analyze that how the composition of the solid electrolyte interphase varies with the salt concentration and with the number of cycles.

## Introduction

Li-metal anode has the potential to increase the energy density of rechargeable batteries thanks to its high volumetric energy density of 2046 mA.h.cm^−3^ and gravimetric energy density of 3862 mA.h.g^−1^. However, the implementation of Li-metal in commercial batteries is hampered by different challenges, in particular by low Coulombic efficiency of plating-stripping and dendrites formation. To overcome these issues, different electrolyte solvents, salts, and additives have been tested^1–3^. In this respect, the concentration of electrolyte salt has been reported to be very important parameter influencing the electrochemical performance of Li-metal anodes^4–7^. For example, Ogumi *et al*. have shown that the performance of Li-metal improved by increasing the salt concentration in propylene (PC) based electrolytes, and this superior performance was referred to the formation of a thinner solid electrolyte interphase (SEI) on Li-metal^4^. The highly concentrated electrolytes -which are often referred to as “solvent in salt” electrolytes- possess unique properties compared to electrolytes with “standard” concentration of 1 M. The high concentration of anions minimizes depletion of anions on the surface of Li-metal, and thus makes the dendrite formation less favourable. The higher concentration could also provide higher ion conductivity and Li^+^ flux, however, this depends on the chemistry of solvent and salt, presence of co-salt, etc., and requires optimization for any electrolyte solution^5,7–9^. The high concentration increases the viscosity of electrolyte solution, which could be beneficial to decrease volatility of electrolytes and to supress the dendrite formation, but brings new challenges for commercial cell manufacturing^5,7^.

A promising work regarding plating-stripping of Li-metal has recently been reported using a concentrated electrolyte of lithium bis(fluorosulfonyl)imide (LiN(SO_2_F)_2_; LiFSI) salt dissolved in 1,2-dimethoxyethane (DME)^8^. The study by Zhang *et al*. indicate that a very stable plating-stripping with high Coulombic efficiency (CE) above 98% for 1000 cycles could be achieved when using high concentration −4 M- of LiFSI in DME. Their results also showed that this high CE was obtained at both low and high current densities comprised between 0.2 mA.cm^−2^ to 10 mA.cm^−2^ for a fixed plating capacity of 0.5 mAh.cm^−2^ ^8^. This superior behaviour of plating-stripping of Li-metal has been suggested to be due to the lower reactivity of electrolyte towards the Li-metal and the formation of a denser SEI layer when the LiFSI salt concentration increased from 1 M to 4 M in DME electrolyte^8,10^. It has also been suggested that LiFSI undergoes complete decomposition and is very efficient to form LiF^11^. Nevertheless, lower CE and cycleability have been reported for 4 M LiFSI in DME electrolyte in other studies^12,13^. Also, Dasgupta *et al*. have shown that a non-dense Li-metal with dendritic structure forms when plating Li-metal in an electrolyte made of 4 M LiFSI in DME while the CE is 93% after only 20 cycles^13^.

The contradictory results shown for Li-metal plating-stripping using LiFSI in DME electrolytes require a new look at both the electrochemical performances of Li-metal and the SEI composition obtained in such electrolytes. Therefore, we here investigate plating-stripping of Li-metal in two different concentrations of 1 M and 4 M LiFSI in DME electrolytes using different current densities and plating capacities. Using X-ray photoelectron spectroscopy (XPS) technique, we also study the influence of the salt concentration on the composition of the SEI formed on Li-metal in the aforementioned electrolytes, and the evolution of the SEI composition with the number of cycles.

## Results and Discussion

**Figure 1:**
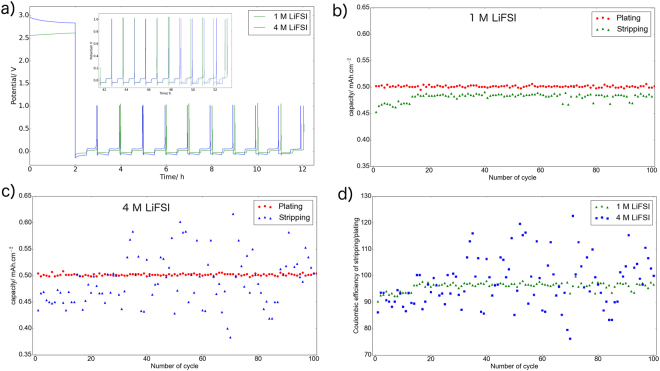
(**a**) Voltage *vs*. time plot of plating-stripping of cycles 1 to 10 in Li|Cu cells using 1 M or 4 M LiFSI in DME electrolytes; the inset shows the results for cycles 40 to 50. A current density of 1 mA.cm^−2^, a constant plating capacity of 0.5 mAh.cm^−2^, and a stripping cut-off potential of 1 V are applied. (**b**,**c**) plating and stripping capacities of each cell, (**d**) Coulombic efficiency of each cell.

Figure 1a shows the electrochemical potential of plating and stripping of Li-metal during the first 10 cycles in the aforementioned electrolytes, and the inset displays the data for cycles 40 to 50 (for clarification, limited number of cycles are shown, while the full cycling data for all 100 cycles can be found in Figure S1). In this experiment, the plating capacity is constant and equals to 0.5 mAh.cm^−2^ in all cycles for both cells, while the stripping is limited by the high cut-off potential of 1 V. Although both cells perform quite similar, the cell with 1 M LiFSI displays slightly smaller overpotential during plating-stripping compared to the cell with 4 M LiFSI. Both cells show quite stable plating capacity, but variations in stripping capacities are observed leading to by CE not close to 100% for both cells, see Fig. 1b–d (CE was calculated by dividing the stripping capacity by the plating capacity). However, the cell with 1 M salt concentration shows stripping capacities closer to 0.5 mAh.g^−1^ compared to the cell with 4 M salt concentration, which highlights that the electrolyte containing a lower concentration of salt provides higher CE; see Fig. 1b–d.

It is worth mentioning that these results are highly reproducible as being representative of the performances of a larger number of cells assembled using the same aforementioned parameters such as electrolytes, current density, plating capacity, etc. and then cycled for different number of cycles; see Figure S2 (these cells were later used for XPS characterization of SEI which is discussed below).

**Figure 2:**
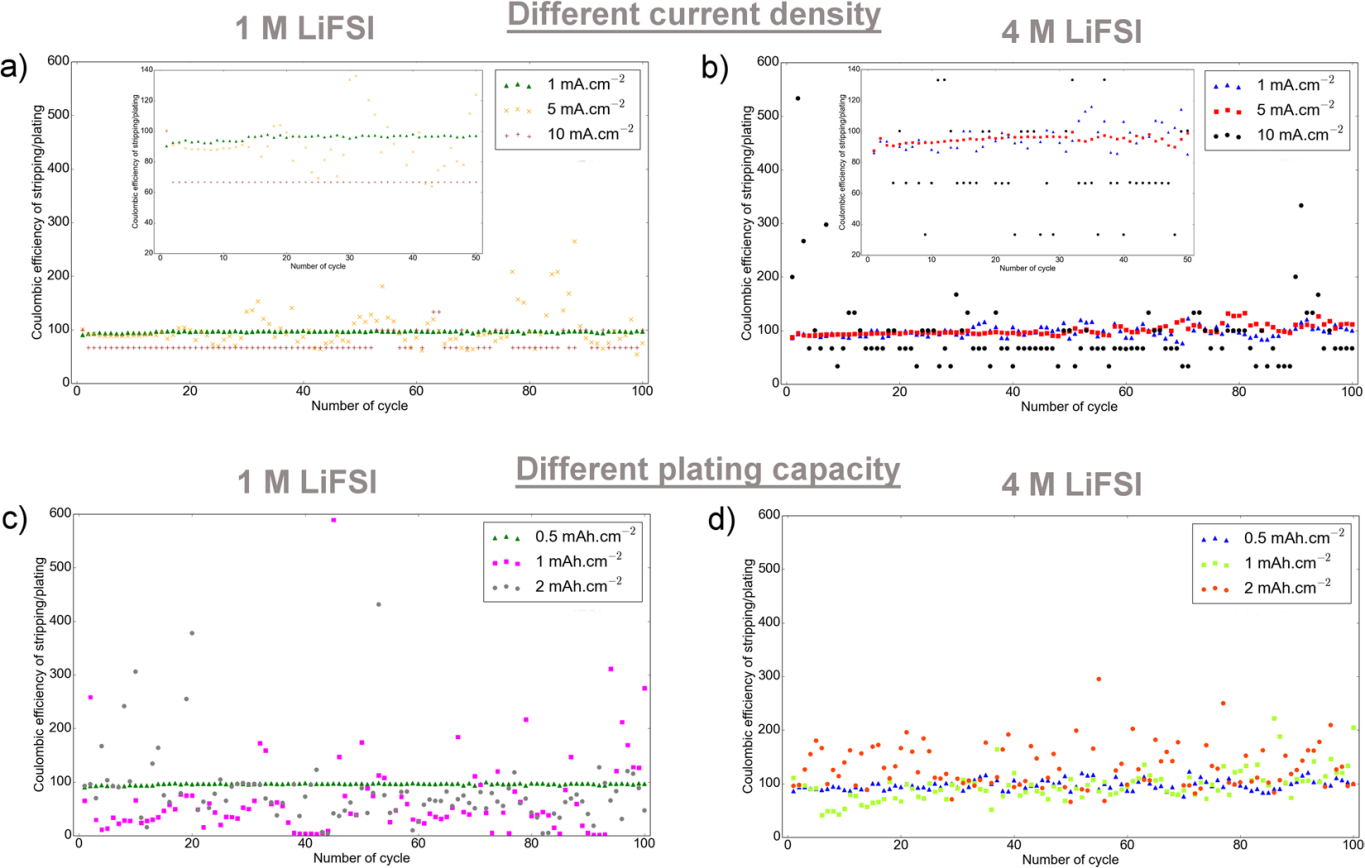
Coulombic efficiency of plating and stripping experiments in Li|Cu cells. (**a**,**b**) Different current densities of 1 mA.cm^−2^, 5 mA.cm^−2^, 10 mA.cm^−2^ are applied while the plating capacity is set to 0.5 mAh.cm^−2^. The data are shown enlarged in the insets. (**c**,**d**) different plating capacities of 0.5 mAh.cm^−2^, 1 mAh.cm^−2^, 2 mAh.cm^−2^ are applied while the current density is set to 1 mA.cm^−2^.

To find out how the applied current density and plating capacity could influence the cell performances, several cells were cycled using different current densities and plating capacities. Figure 2 a–b displays the CE of Li|Cu cells with three different current densities of 1, 5, and 10 mA.cm^−2^. Similar to the previous test, the plating capacity was set to 0.5 mAh.cm^−2^ and the stripping was limited to a high cut-off potential of 1 V. The results show that applying high current densities –for the same plating capacity– result in lower CE for both 1 M and 4 M LiFSI in DME electrolytes. The cells with the current density of 5 mA.cm^−2^ and 10 mA.cm^−2^ have significantly lower CE compared to the cells cycled with 1 mA.cm^−2^. However, with the high current density of 10 mA.cm^−2^ the cell with 1 M LiFSI shows better CE compared to the cell with 4 M LiFSI; the CE is above 60% for low concertation electrolyte while it randomly varies between 30–130% for the high concentration electrolyte. The CE exceeds to above 100% in some cycles for both electrolytes, which indicates that there exist parasitic reactions during stripping step. The origin of these parasitic reactions during stripping could be oxidation of the electrolyte species on the Cu electrodes and/or electrochemical decomposition of SEI components. The results also display that the overpotential increases by100–200 millivolt when the current density increases from 1 mA.cm^−2^ to 10 mA.cm^−2^, and that the overpotential is even higher for the cell with higher concentration electrolyte (see Figure S3).

Figure 2c–d shows the CE of stripping to plating in cells cycled at the same current density of 1 mA.cm^−2^, but for different plating capacities of 0.5 mAh.cm^−2^, 1 mAh.cm^−2^, and 2 mAh.cm^−2^ using the aforementioned electrolytes. The low CE of the cells with 1 and 2 mAh.cm^−2^ plating capacities -compared to the cells 0.5 mAh.cm^−2^ plating capacity- indicate that the performance of Li-metal is quite far from “ideal” in such electrolytes. As expected, the overpotential of plating/stripping observed in the initial cycles is very similar for the cells with different capacities because the current density is the same. Besides this, the cells with higher plating capacity start to malfunction after a lower number of cycles compared to the cells with lower plating capacity, confirming that the amount of Li metal stripped and plated is the main limiting factor for cycling performances. Actually, longer cycling can be obtained if the capacity (amount of Li) is limited. (see Figure S4). On the other hand, the current density –in the tested range in this work- has less influence on cycling performances.

**Figure 3:**
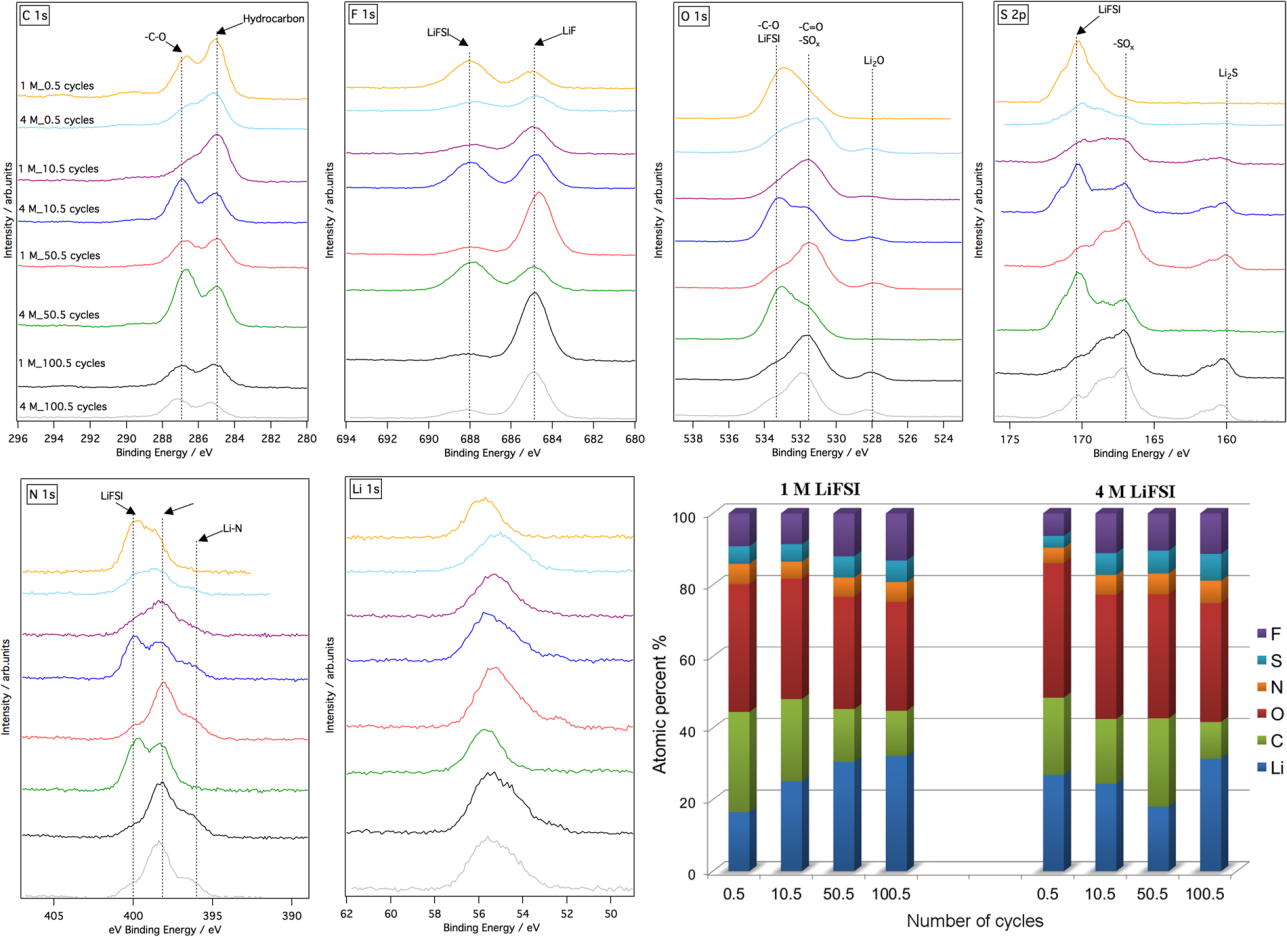
C 1 s, F 1 s, O 1 s, S 2p, N 1 s, and Li 1 s XPS spectra and the relative surface concentration of each element in the SEI formed on plated Li-metal at different number of cycles using electrolytes composed of 1 M or 4 M LiFSI in DME.

To get a deeper understanding on Li-metal plating-stripping in LiFSI in DME electrolytes, the composition of the SEI layer formed on plated Li-metal was characterized by XPS; see Fig. 3. Cells were stopped at different number of cycles in order to investigate whether the composition of the SEI varies with cycles number and with the concentration of LiFSI salt in the electrolyte. Figure 3 displays XPS spectra and the relative surface concentration of each element in the SEI of plated Li-metal after different number of cycles in the aforementioned electrolytes. The spectra indicate that SEI layer formed on plated Li-metal is composed of products of decomposition of both solvent and salt, and reveal that the composition of the SEI varies with the number of cycles and the electrolyte concentration. After half cycle (one plating) the SEI is oxygen rich in both electrolytes. However, after a larger number of cycles, the relative amount O and C decreases while that of F and Li increases in the SEI, which indicates that LiFSI contributes more to the composition of the SEI over long term cycling. F 1 s spectra show that both LiF –formed by decomposition of LiFSI– and LiFSI itself are present in the SEI in both electrolytes, however, the relative amount of LiF increases over long term cycling, indicating that the composition of SEI becomes more inorganic. The other spectra with the assigned peaks^14–16^ shown in Fig. 3 display similar compounds formed in the SEI. The broad Li 1 s spectra with a shoulder on the lower binding energy side suggest that metallic lithium is plated on cupper as detected by XPS. Thus, the thickness of the SEI is expected to be below the sampling depth of the XPS which is about 10 nm. However, on the other hand, the absence of any signal in Cu 2p (see Figure S5) reveals that the total thickness of the SEI and plated Li-metal should be larger than the 10 nm which would result in a blockage of any signals issued from the cupper substrate. The thickness of plated Li-metal could however be estimated assuming the formation of a homogenous Li-metal layer on the Cu substrate with a CE of 100%; it would be equal to almost 2.4 µm for the plating capacity of 0.5 mAh.cm^−2^. As a conclusion, our XPS results suggest that a very thin layer of SEI below 10 nm is formed on top of a thick layer of Li metal. This SEI needs to be highly flexible and stable to cope with huge volume expansion-extraction during the plating-stripping of a relatively thick layer Li-metal, which is indeed a challenging requirement for an SEI layer.

**Figure 4:**
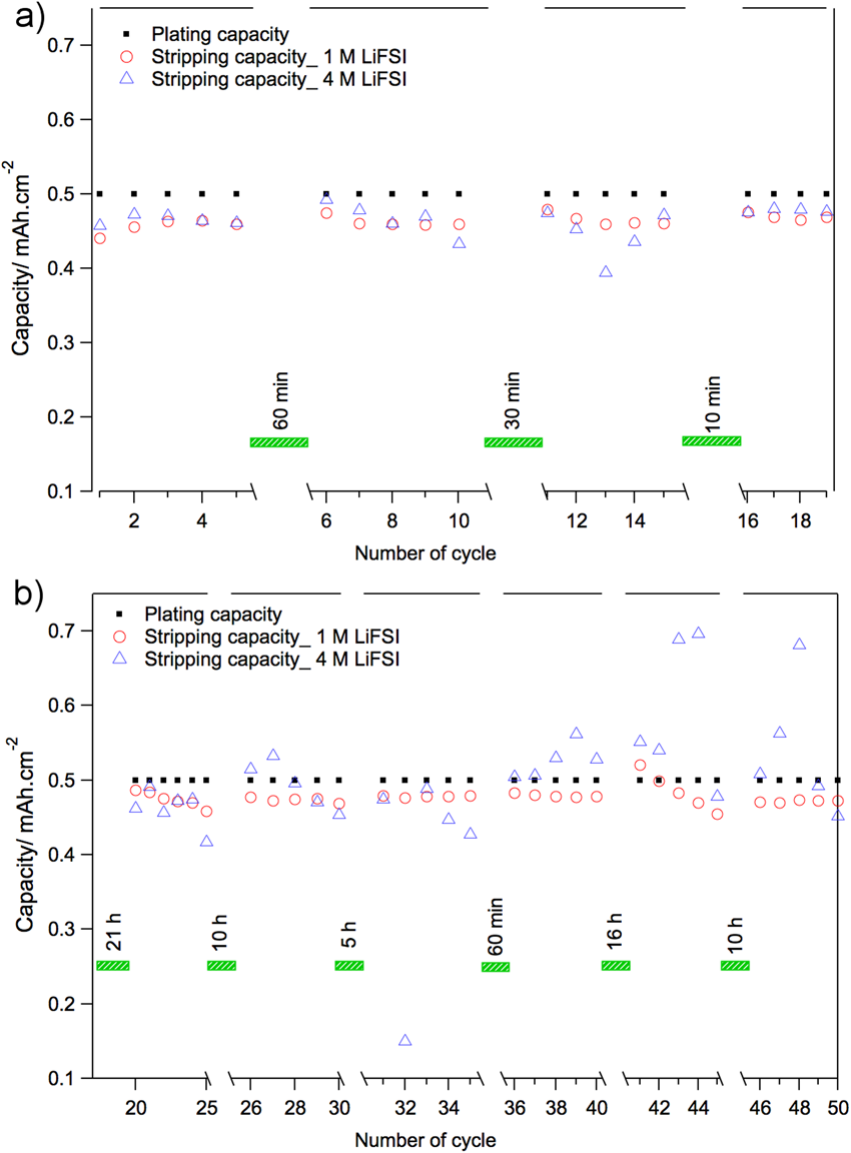
Plating and Stripping capacities of Li-metal with “relaxation” or “pause” time intervals using electrolytes composed of 1 M or 4 M LiFSI in DME. (**a**) cycles number 1 to 19 with pause applied at the stripped state. (**b**) cycles number 20 to 50 with pause time applied at the plated state.

Figure 4 summarizes the plating and stripping capacities obtained for Li|Cu cells stopped at either plated or stripped states for different time intervals with no external current or potential applied (i.e. at open circuit voltage). A capacity loss during the “pause” or “relaxation” time would point out the instability of the SEI layer due to parasitic reactions^17,18^. Figure 4a demonstrates that no major capacity loss occurs when the cells were paused at the stripped state where ideally all Li-metal is stripped off from the cupper substrate. This implies that the SEI is relatively stable and flexible in both electrolytes and remain intact on cupper substrate during volume extraction when Li-metal is removed. Figure 4b shows the plating-stripping capacities for both cells after pause or relaxation time at the plated state. In the cell with 1 M LiFSI, the stripping capacities after different relaxation intervals are close to the plated capacity before the relaxation. Therefore, the plated Li-metal (on cupper) and its SEI are quite stable -and also the formed SEI is passivating- during the relaxation time and no major self-discharge occurred. However, the cell with 4 M LiFSI showed less stability; the stripping capacities after the relaxation were larger than the plated capacities. This means that plated Li-metal and its SEI in the cell with 4 M LiFSI in DME electrolyte are not fully stable during the relaxation, which could be due self-decomposition of electrolyte species on the surface of plated Li-metal durig the relaxation time and consequent electrochemical decomposition of SEI components during the stripping. In other words, the SEI in the cell with 4 M LiFSI is not completely passivating, and therefore, the relaxation time enhances self-decomposition of electrolyte on the surface of plated Li-metal, and part of the formed decomposition products undergo electrochemical oxidation during stripping, resulting to CE above 100%.

In summary, the galvanostatic plating-stripping of Li-metal in Li|Cu cells using LiFSI in DME electrolyte indicate that the CE is not close to 100% for the tested current densities between 1 to 10 mA.cm^−2^ and plating capacities between 0.5 to 2 mAh.cm^−2^. Increasing the concentration of LiFSI from 1 M to 4 M shows no significant improvement in the CE of the Li-metal plating-stripping experiments. Based on a multitude of similar experiments performed to provide repeatability of the present results, it was demonstrated that the higher concentration electrolyte leads to inferior results -at various current densities- when plating capacity equals to 0.5 mAh.cm^−2^. Also, our results highlight that in a given electrolyte concentration, longer cycling can be obtained if the plating capacity is limited while current density –in the range tested in this work- has much less influence on cycling performances. However, we acknowledge that any small variation in the assembly of cells such as impurity in salt^19^, solvent used in cells, cell design^20^, etc. could influence results regarding the plating-stripping of Li-metal. Finally, this study demonstrates the importance of performing systematic studies repeating the same Li plating-stripping experiment several times to insure reproducibility of results irrespective of the cell design or electrolytes studied.

## Methods

### Cell design

Two electrode coin cells (hereafter referred to as Li|Cu) were assembled for plating and stripping experiments. Li metal disks of 15 mm punched out of Li metal sheet (125 µm thick, Cyprus Foote Mineral) and Cu disk of 13 mm in diameter punched out from Cu foil purchased from Goodfellow (Cu000340) with purity of 99.9% and thickness of 20 µm were used as the counter and working electrodes, respectively. Cu disks were soaked in a diluted (∼1 M) HCl for almost 10 min, and then dried in a vacuum furnace before using them in the cells. One layer of Celgard 2325 separator (which is trilayer of polypropylene-polyethylene-polypropylene) dried at 80 °C and 75 µL of electrolyte of LiFSI (1 M or 4 M) in DME were used to assemble cells. The water content of DME (battery grade) purchased from BASF Co. and the prepared electrolytes were determined to be below 4 ppm and 20 ppm, respectively, by Karl-Fischer titration technique. LiFSI with 99% purity was purchased from Suzhou Fluolyte Co., and was dried at 80 °C in a vacuum furnace placed inside an argon-filled glovebox with water and oxygen level below 5 ppm. The electrolyte solutions were prepared by adding required amount of LiFSI salt into DME solvent and leaving it under magnetic stirring overnight.

### Electrochemical test

Galvanostatic discharge-charge tests were performed at room temperature using a DigatronBTS-600. The current density and plating capacity were limited to certain values in different experiments while the stripping capacity was measured when the cell voltage reached 1 V. The Coulombic efficiency was calculated by dividing the stripping capacity by the plating capacity.

### XPS characterization

For XPS measurement, coin cells were stropped at the plated state and then opened in the argon-filled glovebox where electrodes were soaked in DME (battery grade) for almost 1 min to remove the remaining electrolyte. Samples were then transferred to XPS equipment chamber using an air-tight transfer cup filled with Argon. XPS measurements were performed at in-house spectrometer (PHI 5500) using monochromatized Al Kα radiation (1486.6 eV). The XPS spectra were energy calibrated by setting the adventitious carbon peak to 285 eV.

## Electronic supplementary material

Supplementary Information
