# Supplementary material for: Electrochemical performance and interfacial properties of Li-metal in lithium bis(fluorosulfonyl)imide based electrolytes
Source: Sci Rep. 2017 Nov 21;7:15925. doi: 10.1038/s41598-017-16268-7 (PMC5698312; doi:10.1038/s41598-017-16268-7)
Supplement: Supplementary file 1 — Supplementary Information [file 41598_2017_16268_MOESM1_ESM.pdf]

Supplementary Information

for

Electrochemical performance and interfacial properties of Li-metal in lithium bis(fluorosulfonyl)imide based electrolytes

Reza Younesi[1,*], and Fanny Bardé[2]

[1] Department of Chemistry-Ångström Laboratory, Uppsala University, Box 538, Uppsala Sweden

[2] Toyota Motor Europe, Research & Development 3, Advanced Technology 1, Hoge Wei 33 B, Zaventem Zaventem, Belgium

[*] reza.younesi@kemi.uu.se

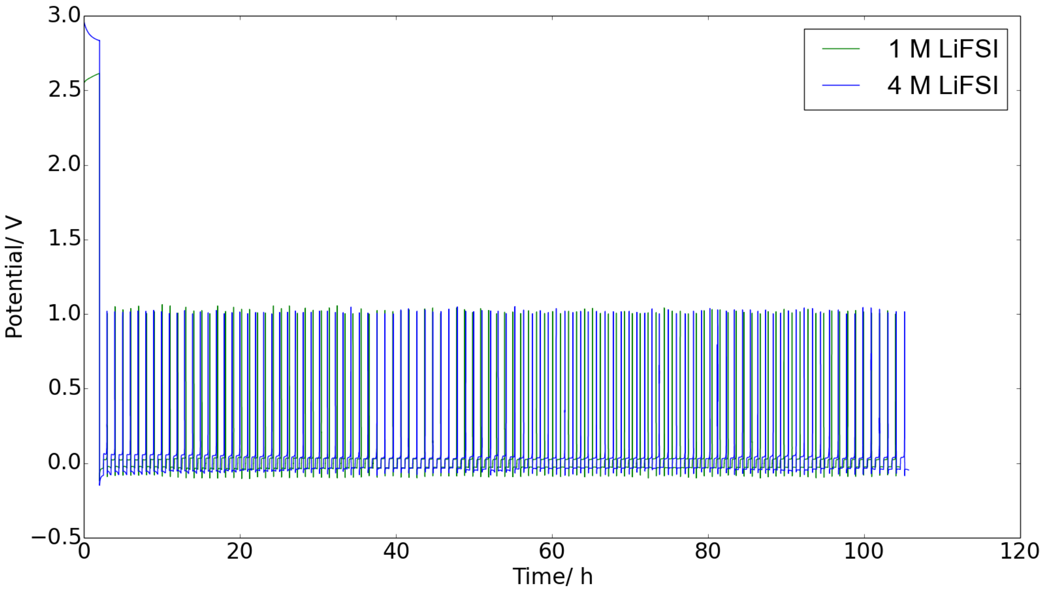

Figure S1. Voltage *vs.* time plot for plating-stripping experiment in Li|Cu cells using 1 M or 4 M LiFSI in DME electrolytes. A current density of 1 mA.cm^{-2}, a constant plating capacity of 0.5 mAh.cm^{-2}, and a stripping cut-off potential of 1 V were applied.

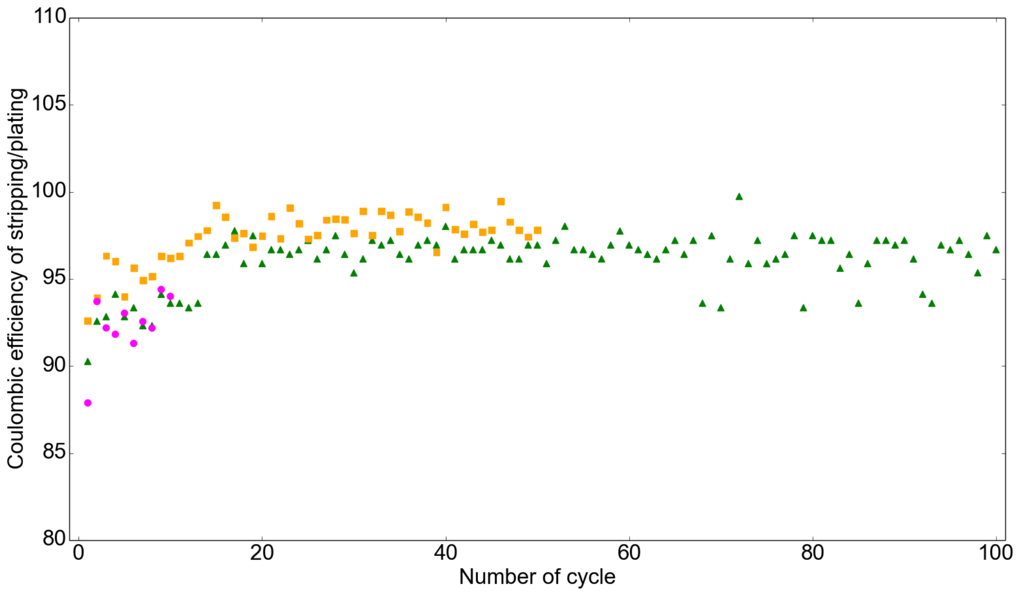

Figure S2. Coulombic efficiency of plating-stripping experiment in 3 different Li|Cu cells using1 M LiFSI in DME electrolyte, and applying a current density of 1 mA.cm^{-2}, and a plating capacity of 0.5 mAh.cm^{-2}. The cells were stopped at different cycles number for XPS characterization of the SEI.

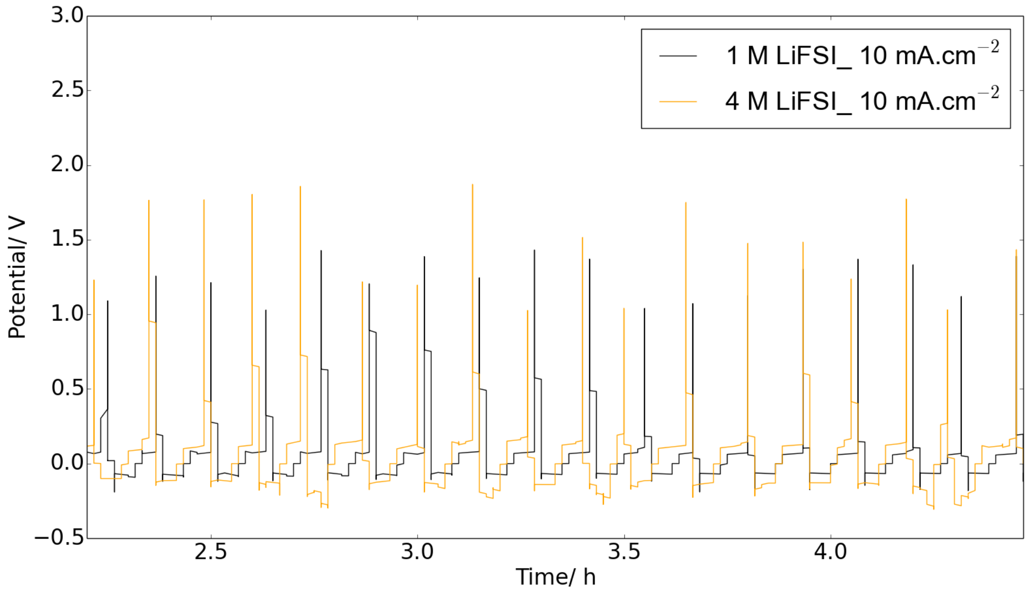

Figure S3. Voltage *vs.* time plots of plating and stripping experiments in Li|Cu cells using 1 M or 4 M LiFSI in DME electrolyte at a current density of 10 mA.cm^{-2}.

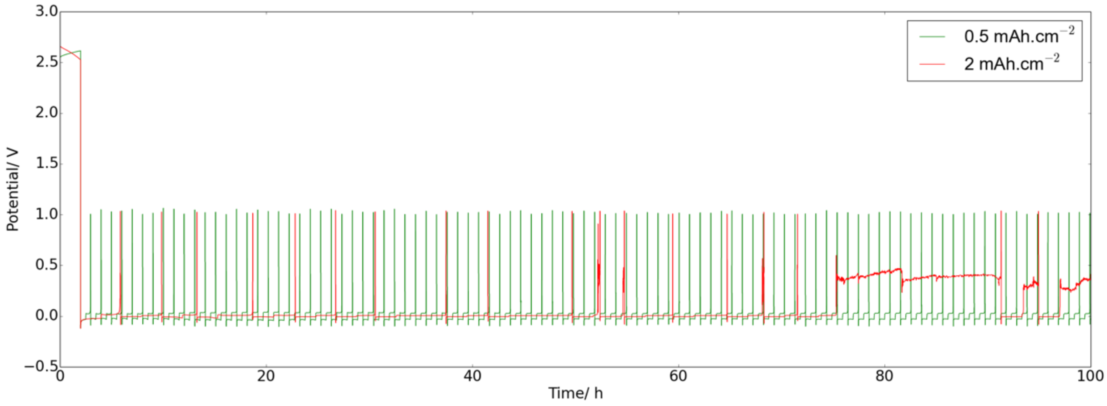

Figure S4. Voltage *vs.* time plots of plating and stripping experiments in Li|Cu cells using 1 M LiFSI in DME electrolyte, at a current density of 1 mA.cm^{-2}and for different plating capacities.

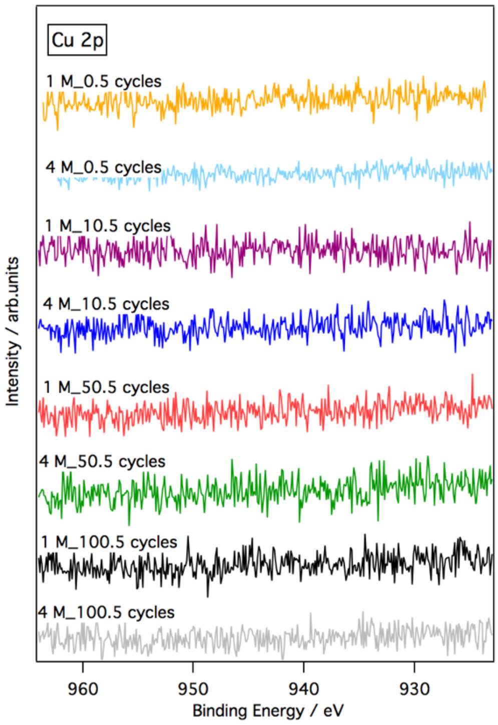

Figure S5. Cu 2p spectra of plated Li-metal on cupper susbtrate in Li|Cu cells at different cycles number using electrolytes composed of 1 M or 4 M LiFSI in DME.
